# Determinants of poor prognosis after surgery for glioblastoma: A retrospective analysis of predictors of morbidity and mortality in a low-middle income country

**DOI:** 10.12669/pjms.41.13(PINS-NNOS).13364

**Published:** 2025-12

**Authors:** Sundas Irshad, Haseeb Mehmood Qadri, Ahsan Sarwar, Mahroona Fatima Khalid, Omair Sajjad, Muhammad Jamil, Muhammad Hannan Tayyab, Asif Bashir

**Affiliations:** 1 Sundas Irshad, MBBS, Postgraduate Resident, Department of Neurosurgery, Punjab Institute of Neurosciences, Lahore, Pakistan; 2 Haseeb Mehmood Qadri, MBBS,Postgraduate Resident, Department of Neurosurgery, Punjab Institute of Neurosciences, Lahore, Pakistan; 3 Ahsan Sarwar, MBBS,Post Graduate Resident, Department of Neurosurgery, Punjab Institute of Neurosciences, Lahore, Pakistan; 4 Mahroona Fatima Khalid, MBBS, Postgraduate Resident, Department of Neurosurgery, Punjab Institute of Neurosciences, Lahore, Pakistan; 5 Omair Sajjad, MBBS, FCPS,Senior Registrar, Department of Neurosurgery, Punjab Institute of Neurosciences, Lahore, Pakistan; 6 Muhammad Jamil, MBBS,Postgraduate Resident, Department of Neurosurgery, Punjab Institute of Neurosciences, Lahore, Pakistan; 7 Muhammad Hannan Tayyab, MBBS, FCPS,Senior Registrar, Department of Neurosurgery, Punjab Institute of Neurosciences, Lahore, Pakistan; 8 Asif Bashir, MBBS, MD, FACS. FAANS,Diplomat American Board of Neurosurgery, Professor and Head of Department Neurosurgery, Punjab Institute of Neurosciences, Lahore, Pakistan

**Keywords:** Glioblastoma, Glioma, Karnofsky performance status, Morbidity, Treatment outcome

## Abstract

**Background & Objective:**

High-grade gliomas (HGG) and glioblastomas are the most prevalent types of primary brain tumors. Despite aggressive treatment, recurrence is almost inevitable with significantly poor prognosis. We aimed to determine the factors associated with morbidity and mortality in patients with glioblastoma after surgical excision.

**Methodology:**

In this retrospective observational study, data regarding patient demographics, clinical features, tumor characteristics and treatment outcomes was recorded for all patients who underwent glioblastoma multiform tumor excision surgery at Punjab Institute of Neurosciences between 2022 and 2024. Multiple predictor variables were identified using univariate and multivariate analysis. The relationship between overall survival and predictor variables was presented in the form of Kaplan Meir survival curves. Data was analyzed using SPSS version 29.

**Results:**

The mean age of study participants was 47.0±15.8 years with 82% (41) males. The mean preoperative Glasgow coma scale (GCS) and Karnofsky Performance Status (KPS) was 13.2±2.8 and 65.2±17.6 respectively. The medial overall survival was 381.5 days. Obesity, tumor origin, preoperative and post-operative GCS and KPS score, and extent of resection were identified as significant predictor variables using univariate analysis (p<0.05). On multivariate analysis, extent of resection was a significant positive predictor of survival, HR=0.041 (95% CI: [0.003-0.581]).

**Conclusion:**

Majority of gliomas undergo gross total resection and had a median survival of one year. Extent of resection is a significant independent predictor of survival in glioblastomas, where gross total resection is associated with a lower risk of mortality.

## INTRODUCTION

Gliomas are the most common type of malignant primary brain tumor. In this category, glioblastomas are the most prevalent.^1^ Glioblastoma is the most common form of primary brain cancer, making up approximately 54% of glioma cases.^2^ Almost three to four people per 100,000 get affected and has a median survival rate of 15 months. Patients are diagnosed between the ages of 59-65, with older adults being more vulnerable. Surgery has a critical role in treating HGG, to carry out a biopsy or gross total tumor excision as part of the treatment plan.^1^

Primary glioblastoma is more common in men while secondary glioblastoma is more frequently diagnosed in women. In terms of glioma classification, Grade-I tumors have a low growth rate and can often be treated surgically. In contrast, Grade-II to Grade-IV gliomas is highly aggressive and malignant. Alarmingly, they are the third leading cause of cancer deaths among young individuals aged 15-34.^1^-^4^

Glioblastomas typically occur in the frontal, temporal, parietal, and occipital lobes, as well as other brain structures. They usually present with headache, seizures, motor deficit, neurocognitive impairment, gait disturbance and personality disorders, depending upon location of lesion.^5^ Standard treatments for newly diagnosed glioblastoma patients involves surgery, tumor resection, and postoperative radiotherapy, combined with temozolomide therapy. Despite aggressive treatment, recurrence is almost inevitable, with most patients experiencing tumor recurrence within 32-36 weeks after initial therapy. Fortunately, ongoing advances in genetic and molecular research offer hope for improved management and outcomes for this aggressive tumor, potentially paving the way for more effective treatments in the future.^2^,^3^,^4^,^6^ However, the impact of genetic mutations and molecular profiling over patient prognosis have been rarely studies in Pakistan owing to unavailability of molecular testing in public sector hospitals.^7^ A recent study by Bjawa et al. reported the treatment patterns of gliomas in Pakistan, yet it was a simple epidemiological study with evidence regarding impact of patient variables on patient prognosis and mortality.^8^ Another study by Wadd et al. studied prognostic factors of glioblastoma yet age was the only demographic factors studied apart from clinical parameters.^9^

The causes of death from glioblastoma and other aggressive brain tumors are multifaceted, influenced by various factors such as underlying health conditions and complicated clinical circumstances. Unfortunately, according to the literature search conducted on PubMed central, the factors affecting morbidity and mortality of glioblastoma have not been studied in lower middle income countries especially in Pakistan. Therefore, this study aimed to thoroughly investigate the causes of death, as well as the use of palliative care and life-saving measures, in glioma patients at a major academic medical center.^6^

## METHODOLOGY

This was a retrospective observational study conducted in Department of Neurosurgery, Punjab Institute of Neurosciences, Lahore between 2022 and 2024. All the patients who underwent surgical treatment of glioblastoma excision were included. Study sample was calculated using non-probability convenient sampling technique. Data was collected regarding patient demographics, radiological data, operative notes, clinical notes, post-operative stay in the hospital, post-op clinic follow-up, and rehabilitation/recovery. Depending on the availability of data, outcomes were assessed at immediate post-operative, three months (±four weeks), six months (±four weeks) and annual follow-up. The study was approved by Institutional Review Board of Punjab Institute of Neurosciences with reference number 2038/IRB/PINS/Approval/2025; dated January 23, 2025.

### Inclusion criteria

- All patients with glioblastoma, aged 18 years or above, who underwent surgical resection with at least three-month follow-up data available were included.

### Exclusion criteria

- Patients with incomplete data.
- Patients who were managed conservatively.

### Data analysis:

The data was analyzed using SPSS version 29 (IBM Corp. Released 2023. IBM SPSS Statistics for Windows, Version 29.0.2.0 Armonk, NY: IBM Corp). Means and standard deviations were calculated for continuous variables and frequencies and percentages were calculated for categorical variables. The comparison between baseline characteristics was done using Fischer’s Exact test for categorical variables and Mann-Whiteny U test for continuous variables. Gross total resection (GTR) was defined as removal of 90–99% of the tumour mass, while partial tumour resection was defined as <90% resection on postoperative MRI. Overall survival (OS) was defined as duration of time from surgical intervention until death or last follow-up. Socioeconomic status was stratified as high and low on the basis of occupation according to Bajwa et al.^8^ employing Pakistan Bureau of Statistics guidelines.^10^ A univariate survival analysis using the Kaplan Meier method with the log-rank test was conducted to identify predictor variables. Principle Component analysis was used to refine these variables and remove collinearity. Multivariable analysis was done on all factors with maximum factor loading in each component identified by principal component analysis. The relationship between overall survival and predictor variables was presented in the form of Kaplan Meir survival curves.

## RESULTS

A total of 50 patients were studied who had undergone glioblastoma surgery at our institute. Out of these 50 patients, 76% (38) patients had gross total resection (GTR) of tumor done while 24% (12) (patients underwent partial resection. The mean age of study participants was 47.0±15.8 years with 82% (41) males and 18% (9) females. Among them, 70% (35) were urban residents, 82% (41) patients belonged to lower socioeconomic status, 64% (32) had hypertension, 52% (26) had diabetes, 14% (7) were smokers, 2% (1) had ischemic heart disease, 2% (1) were obese. The mean preoperative GCS was 13.2±2.8 and postoperative GCS was 12.8±3.6. Similarly, the mean preoperative KPS score was 65.2±17.6 whereas mean postoperative KPS score was 52.4±26.1. Two patients in our study received neo-adjuvant treatment, one received radiotherapy for four months and the other underwent cyber knife radiosurgery with no record available. The demographic and baselines characteristics of study participants stratified according to the extent of resection are given below (Table-I).

**Table-I:** Baseline characteristics of study participants.

Characteristics	Total Resection (>90%)	Partial Resection (<90%)	p-value*
No of participants, N	38	12	-
Age, Mean±S.D	46.2±16.1	49.4±15.0	0.601
***Age Group***			1.000
≤55 years	24 (63.2)	7 (58.3)	
>55 years	14 (36.8)	5 (41.7)	
***Gender***			0.668
Male	32 (84.2)	9(75)	
Female	6 (15.8)	3 (25)	
***Type of residence***			1.000
Urban	26 (68.4)	9(75)	
Rural	12 (31.6)	3 (25)	
***Occupation***			0.720
Laborers	13 (34.2)	5 (41.7)	
Businessman	5 (13.2)	2 (16.7)	
Farmer	5 (13.2)	0 (0)	
Others	15 (39.5)	5 (41.7)	
***Comorbidities***			
Hypertension	24 (63.2)	8 (66.7)	1.000
Diabetes	19 (50)	7 (58.3)	0.745
Smoking/Huqqa	6 (15.8)	1 (8.3)	1.000
Ischemic Heart disease	1 (2.6)	0 (0)	1.000
***BMI (kg/m^2^)***			0.240
>30	0 (0)	1 (8.3)	
<30	38 (100)	11 (92.7)	
Use of Cocaine or illicit drugs	0 (0)	0 (0)	
Use of Antihypertensive or Antidiabetic drugs	4 (10.5)	0 (0)	1.000
Family history	1 (2.6)	0 (0)	
Preoperative CSF diversion	0 (0)	1 (8.3)	0.240
Intracranial Hypertension	37 (97.4)	0 (0)	1.000
***Case Status***			1.000
1^st^ time surgery	37 (97.4)	(0)	
Recurrent case	1 (2.6)	(0)	
***GCS***			
Preoperative	13.6±2.1	12.0±4.2	0.435
Postoperative	13.5±2.5	10.7±5.6	0.272
***KPS***			
Preoperative	70 (20-80)	75 (20-90)	0.925
Postoperative	65 (0-80)	15 (0-80)	0.155
Neoadjuvant Treatment	2 (5.3)	0 (0)	1.000

* Fischer’s exact test.

A total of seven predictor variables including obesity, tumor origin, preoperative and post-operative GCS and KPS score, and extent of resection were identified using univariate analysis for cox hazard proportional model (i.e., p<0.05 for univariate analysis) (Table-II).

**Table-II:** Univariate Analysis of Predictor variables using Kaplan Meir analysis and Log rank test.

Variable	Log-Rank	Df	p-value
Age (<55, >55)	0.061	1	0.805
Gender (males, females)	0.000	1	0.984
Type of residence (urban, rural)	0.025	1	0.874
Occupation (Worker, businessman, farmer, others)	2.08	3	0.555
Socioeconomic status (high/low)	1.54	1	0.213
Hypertension (yes, no)	0.773	1	0.379
Diabetes (yes, no)	0.411	1	0.521
Smoking/Huqqa (yes, no)	0.07	1	0.934
Ischemic heart disease (yes, no)	0.140	1	0.708
Obesity (yes, no)	9.951	1	0.002*
Use of antihypertensives/antidiabetics (yes, no)	0.602	1	0.438
Neoadjuvant Treatment (yes, no)	0.287	1	0.592
Diagnosis (Frontal, parietal, temporal, insular, anterior skull base, brainstem, overlapping)	4.510	1	0.608
Tumor origin (frontal, temporal, parietal, basal ganglia, brainstem, overlapping)	15.5	5	0.008*
Tumor location (anterior cranial fossa, middle cranial fossa, posterior cranial fossa, mixed)	1.024	3	0.795
Tumor laterality (left, right, midline, bilateral)	0.604	3	0.896
Case status (1^st^ surgery, recurrent surgery)	0.140	1	0.708
Preoperative GCS (3-7, 8-12, 13-15)	13.6	2	0.001*
Postoperative GCS (3-7, 8-12, 13-15)	56.609	2	<0.001*
Preoperative KPS (0-40, 50-70, 80-100)	5.766	2	0.056*
Postoperative KPS (0-40, 50-70, 80-100)	513.09	2	0.001*
Extent of Resection (total resection, partial resection)	14.8	1	<0.001*

* statistically significant.

Principal component analysis yielded only three predictor variables for multivariate analysis including tumor origin, preoperative KPS score, and extent of resection based on highest factor loadings in each component (Table-III).

**Table-III:** Principal Component Analysis.

Variable	Component 1	Component 2	Component 3
Obesity	-0.171	-0.685	-0.089
Tumor Origin	-0.118	0.045	0.938
Preoperative GCS	0.645	0.542	-0.196
Postoperative GCS	0.425	0.691	0.285
Preoperative KPS	0.842	0.025	-0.132
Postoperative KPS	0.771	0.184	0.080
Extent of resection	0.115	-0.739	0.475

Multivariable analysis examined the association between tumor origin, preoperative KPS score and extent of with survival time. The results indicated that extent of resection was a significant predictor of survival (B=−3.194, SE=1.352, Wald=5.579, p<0.05). The hazard ratio HR=0.041 (95% CI: [0.003-0.581]) suggests that gross total resection was associated with a lower risk of mortality. The model fit was significant, χ2(6) =17.8, p<.001, indicating that the predictor explained a significant portion of the variance in survival time. Contrary to this, the results of multivariate analysis were not significant for preoperative KPS score and tumor origin (p<0.05) (Table-IV).

**Table-IV:** Multivariate Analysis using Cox regression model.

Predictor variable	HR	95% CI (lower-upper)	p-value
***Tumor Origin***			
Category 1	0.449	0.024-8.211	0.589
Category 2	0.314	0.023-4.202	0.381
***Preoperative KPS***			
Category 1	0.442	0.032-6.185	0.545
Category 2	0.246	0.018-3.320	0.291
Extent of resection	0.041	0.03-0.581	<0.05*

* Statistically significant.

**Fig.1:**
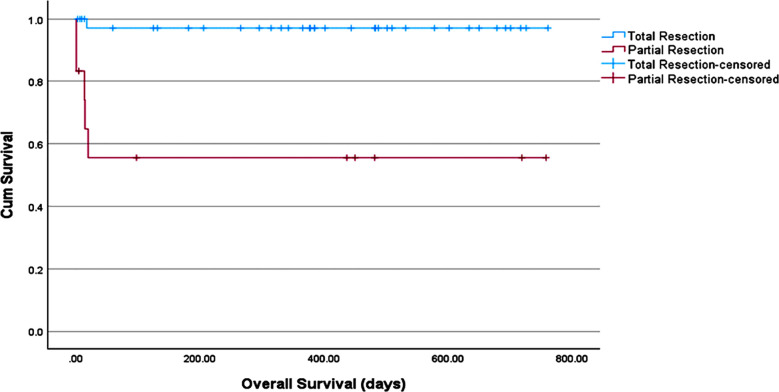
Kaplan-Meir Survival curve showing overall survival in GBM categorized based on extent of resection.

The median overall survival was 381.5 days. The average follow up time was 437 days (95% CI: 359.8-514.1), estimated using reverse Kaplan Meir method. The Kaplan-Meir analysis curves for overall survival categorized on the basis of extent of resection is given below (Fig.1).

## DISCUSSION

This study proposed obesity, tumor origin, preoperative and post-operative GCS and KPS score and extent of resection as significant prognostic predictors of glioblastoma on univariate analysis yet, extent of resection was the only significant independent prognostic factor that determined the overall survival in patients with glioblastoma on multivariate analysis, while no effect of socioeconomic status on patient survival was observed. Additionally, the results revealed an overall increased occurrence of glioblastoma in males, younger age group and urban population.

Almost 82% (41) of the patients in our study were males. This is supported in literature by Grochans et al. who reported an increased incidence of glioblastoma in males, which may occur due to lack of protective effect of female hormones and tumorigenic effect of male hormones.^11^ Similarly, an increased occurrence of glioblastoma in urban population was also observed by Ostrom et al.^12^ Yet the logical reasoning behind this increased incidence is still an ambiguity, with increased chemical exposure, ionizing radiations and early diagnosis as one of the stated yet not definitive reasons.^12^ Moreover, the association of type of residency with overall survival of patients with glioblastoma as seen in literature was not observed in our study.^13^

Unlike, Brown et al. who reported younger age as a significant independent positive prognostic factor for glioblastoma, no significant differences in overall survival were observed between younger and elderly age group, despite the fact that almost 62% (31) of study participants were adults, of which 76% (38) underwent GTR, a proposed reason for overall improved survival in younger patients.^14^

Females with glioblastoma are shown to have an overall improved survival in comparison to males. The gender disparity associated with good prognosis of glioblastoma, as described previously in literature in the form overall survival benefit in females, is not observed in our study.^14^ The improved overall survival in female patients with glioblastoma may occur due to gender-based variations in genetic expression and methylation patterns (for instance, NOX, FRG1BP, PUDP, SYAP1 KDM6A, and DDX3X), an increased tendency of male astrocytes for malignant transformation in comparison to female astrocytes and protective role of gonadal steroid hormones, yet these gender based differences were not observed in our study, perhaps due to smaller representation of females in our data, comprising of only 18% (9) of the sample.^11^,^15^,^16^

Obesity was found to have significant poor prognostic significance on univariate analysis, implying an overall improved survival in patients with normal BMI (i.e., BMI<30). This is parallel to the findings of another study by Cappelli et al. who reported that normal BMI (19-24.99) is associated with good overall survival and progression free survival in glioblastoma patients.^17^ This might be attributed to the fact that obesity leads to insulin resistance and raised IGF-1 levels which inhibits apoptosis. Contrarily, Naik et al. has also proposed the paradoxical protective effect of obesity on tumor progression and survival.^18^

Similar to the previously existing literature, tumor origin had prognostic significance on univariate analysis.^19^,^20^ According to Fyllingen et al. glioblastoma arising from central brain including corpus callosum, basal ganglia, and left temporal lobe had a less overall survival in comparison to tumors originating from rest of the brain.^19^ Additionally, variable genetic biomarker expression in different hemispheres may also lead to differences in overall survival in tumors originating from different locations of brain.^20^

The results revealed an overall higher survival in patients with good preoperative and postoperative KPS score although the results were not significant on multivariate analysis, implying that KPS score is not an independent predictor of prognosis for glioblastoma. A study by Gunawan et al. reported an overall improved follow up outcomes at two months in patients with good preoperative KPS.^21^ The median preoperative KPSs in our study was 70% before surgery and 60% after surgery, with the difference in KPS is similar to the one provided by Gunawan et al. i.e. 60% and 50% respectively.^21^ Chambless et al. also stated the superior predictive ability of postoperative KPS in determining the long-term outcomes of glioblastoma in comparison to preoperative KPS.^22^ Contrarily, the prognostic role of GCS in determining overall survival of patients, seen in our univariate analysis, is not observed in literature, as most of the researchers relied upon KPS for determining prognosis rather than GCS, which is an effective marker of consciousness, rather than functionality of patients as required in oncological follow-ups.^23^

Considering the impact of neurosurgical intervention, the study revealed an overall significantly improved survival in patients who underwent GTR (>90%). This is supported in literature by Skardley et al.^24^ who reported an overall decrease in survival time by 0.88 with each 10cm^3^ residual tumor volume after surgical resection in glioblastoma, yet the variability in grading of extent of resection is still a cause of controversy yielding variable cutoff values for EOR with different survival outcomes.^25^,^26^ Both IDH mutation and MGMT promoter methylation are also reported to affect overall survival in patients with glioblastoma, yet the local literature is from private institutions where patient affordability is not an issue.^27^-^30^

### Limitations:

Although the study provides a deep insight into the prognostic factors which affect the survival of patients with glioblastoma, the findings are limited owing to small sample size, single institution-based data, retrospective study design, lack of data regarding genetic mutations, molecular profiling of glioblastoma patients and postoperative adjuvant therapy which is known to significantly influence patient survival.

## CONCLUSION

Glioblastomas are common CNS tumors with a poor prognosis commonly dealt by GTR. The overall survival is worse in obese patients, patients with variable tumor origin, poor pre- and post-op GCS and KPS, and partially resected tumors. Extent of resection is the only independent predictor of overall survival. Age and gender generally do not impact overall survival. Neo-adjuvant and adjuvant chemoradiotherapy along with steroids administration might slightly prolong the average life span.

### Clinical recommendations:

The findings of our study recommend the need of considering maximal safe resection in improving patient outcomes in glioblastoma. Moreover, incorporating data regarding genetic and molecular profiling of patients along with postoperative adjuvant therapy in future studies will further clarify the prognosis of various types of glioblastoma along with their responsiveness to chemoradiotherapy. Thus, helping neurosurgeons in improved decision making.

### Authors’ Contributions:

**SI:** Data interpretation and critically reviewed the manuscript

**HMQ:** Concept and Design of the study, critical review of the manuscript and supervision.

**AS, MFK & MJ:** Data acquisition and drafted the manuscript.

**OS MHT AB:** Data interpretation and critically reviewed the manuscript.

All the authors have read and approved the final manuscript and are responsible and accountable for the accuracy and integrity of the work.
